# Inference of a Geminivirus−Host Protein−Protein Interaction Network through Affinity Purification and Mass Spectrometry Analysis

**DOI:** 10.3390/v9100275

**Published:** 2017-09-25

**Authors:** Liping Wang, Xue Ding, Jiajing Xiao, Tamara Jiménez-Gόngora, Renyi Liu, Rosa Lozano-Durán

**Affiliations:** 1 Shanghai Center for Plant Stress Biology, Chinese Academy of Sciences, Shanghai 201602, China; wlp@sibs.ac.cn (L.W.); dingxue@sibs.ac.cn (X.D.); jjxiao@sibs.ac.cn (J.X.); tamara@sibs.ac.cn (T.J.-G.); 2 University of the Chinese Academy of Sciences, Beijing 100049, China

**Keywords:** Geminivirus, TYLCV, *Nicotiana benthamiana*, protein-protein interactions, network, hubs, broad-spectrum resistance, pathogens, plant, host

## Abstract

Viruses reshape the intracellular environment of their hosts, largely through protein-protein interactions, to co-opt processes necessary for viral infection and interference with antiviral defences. Due to genome size constraints and the concomitant limited coding capacity of viruses, viral proteins are generally multifunctional and have evolved to target diverse host proteins. Inference of the virus-host interaction network can be instrumental for understanding how viruses manipulate the host machinery and how re-wiring of specific pathways can contribute to disease. Here, we use affinity purification and mass spectrometry analysis (AP-MS) to define the global landscape of interactions between the geminivirus *Tomato yellow leaf curl virus* (TYLCV) and its host *Nicotiana benthamiana*. For this purpose, we expressed tagged versions of each of TYLCV-encoded proteins (C1/Rep, C2/TrAP, C3/REn, C4, V2, and CP) in planta in the presence of the virus. Using a quantitative scoring system, 728 high-confidence plant interactors were identified, and the interaction network of each viral protein was inferred; TYLCV-targeted proteins are more connected than average, and connect with other proteins through shorter paths, which would allow the virus to exert large effects with few interactions. Comparative analyses of divergence patterns between *N. benthamiana* and potato, a non-host *Solanaceae*, showed evolutionary constraints on TYLCV-targeted proteins. Our results provide a comprehensive overview of plant proteins targeted by TYLCV during the viral infection, which may contribute to uncovering the underlying molecular mechanisms of plant viral diseases and provide novel potential targets for anti-viral strategies and crop engineering. Interestingly, some of the TYLCV-interacting proteins appear to be convergently targeted by other pathogen effectors, which suggests a central role for these proteins in plant-pathogen interactions, and pinpoints them as potential targets to engineer broad-spectrum resistance to biotic stresses.

## 1. Introduction

Viruses reshape the intracellular environment during infection, both to co-opt processes necessary for the development of the viral infection and to interfere with antiviral defenses. Studies of virus-host interactions have proven instrumental for understanding how viruses manipulate the host machinery, and have provided a wealth of insight into the biology of the host cell, including how re-wiring of specific pathways can contribute to disease. Due to genome size constraints and concomitant protein coding limitations, viral proteins have evolved to achieve multifunctionality, partly through targeting diverse host proteins. The number of interactions of a viral proteome is therefore anticipated to be substantial, as indicated by recent high-throughput proteomics analyses of virus-host protein-protein interactions in mammalian cells [1,2,3,4,5,6] and in plants [7]. Systems-level analyses can identify pathways or proteins that are main targets of viruses [8,9], which would in turn make good targets for anti-viral strategies. In animal viruses, systems biology approaches have been applied to successfully uncover strategies used by the RNA viruses human immunodeficiency virus (HIV) and hepatitis C virus (HCV), and the DNA virus Kaposi’s sarcoma herpesvirus (KSHV), using affinity tag purification and mass spectrometry (AP-MS) [1,2,3,5]. In plants, AP-MS has been recently applied to define the interactome of the NIa protein from the potyvirus *Tobacco etch virus* [7]. 

Viruses and other pathogens have been shown to target proteins that play a central role in the modular organization of protein interaction networks [10,11,12,13], also known as hub proteins, in both animals and plants [3,14,15,16,17]. Hub proteins may be more conserved than non-hub proteins due to evolutionary constraints [18]. Interestingly, pathogen effectors from three different kingdoms of life (oomycetes, fungi, and bacteria) have been shown to convergently target plant proteins, many of which are hubs [15,17]. 

Geminiviruses, insect-transmitted plant viruses with circular single-stranded DNA genomes, cause some of the most economically important diseases in major vegetable and field crops worldwide, including cassava, cotton, maize, bean or tomato [19,20]. Therefore, geminiviruses pose a major threat to sustainable agriculture and food security at a global scale. Moreover, in the past few decades geminiviruses have extensively proliferated, their dispersion likely exacerbated by movement through commerce of plant products around the globe and by the rise in temperature due to global warming, which favours the spread of their insect vectors. Understanding the molecular mechanisms underlying pathogenicity and dispersion of geminiviruses is a crucial step towards the design of effective strategies for crop protection. Although geminiviruses have very simple genomes, with only four to eight genes, the detailed molecular events in their interactions with host plants are poorly understood. Because of their small genomes, geminiviruses are an ideal model system to study the plant/virus interface by defining the global interactome of viral proteins in the host cell. Importantly, this approach would not only shed light on the cellular and molecular mechanisms underlying the viral infection, but could also lead to the identification of valuable targets for breeding resistance.

In this work, we investigate the global landscape of interactions between the geminivirus *Tomato yellow leaf curl virus* (TYLCV) and the host *Nicotiana benthamiana* through in planta expression of tagged viral proteins, in the context of the viral infection, followed by AP-MS. The results obtained through this approach were used to infer a network of TYLCV-*N. benthamiana* interactions, comprising six viral proteins and 728 host proteins. Network-based analysis revealed that TYLCV targets highly connected host proteins, or hubs, which would potentially result in larger effects on multiple cellular functions. Remarkably, some TYLCV-interacting proteins appear to be convergently targeted by effectors from fungi and oomycetes, which suggests an essential role of these proteins in plant biotic interactions and identifies them as potential targets to engineer broad-spectrum resistance to pathogens.

## 2. Materials and Methods

### 2.1. Plasmids and Cloning

Open reading frames (ORFs) from TYLCV (GenBank accession number AJ489258) corresponding to Rep, C2, C3, C4, V2, and CP, were polymerase chain reaction (PCR)-amplified and cloned in pENTR-D/TOPO (Invitrogen) with or without a stop codon, and verified by Sanger sequencing. Primers used in this work are listed in Supplementary Table S4. TYLCV genes were then gateway-cloned into the binary vectors pGWB5 and pGWB6 [21] to generate green fluorescent protein (GFP) fusion proteins at the C- or the N-terminus, respectively.

### 2.2. Affinity Purification-Mass Spectrometry Analysis

AP-MS was performed as described in [22]. In brief, viral proteins were transiently expressed in four-week-old *N. benthamiana* leaves by agroinfiltration. Samples were taken two days post-infiltration; accumulation of the GFP-fused proteins was confirmed by confocal microscopy. After grinding the plant tissue in liquid nitrogen, total proteins were extracted by adding lysis buffer (100 mM Tris-HCl pH 8.0; 150 mM NaCl; 10% glycerol; 5 mM EDTA; 5mM DTT, 1 mM PMSF; 1% protease inhibitor cocktail; 2% NP-40) and the extracts were cleaned by filtration; extracts were incubated with GFP-Trap beads (Chromotek, Germany) for one hour, and beads were subsequently washed using washing buffer with detergent (100 mM Tris-HCL pH 8.0; 150 mM NaCl; 10% glycerol; 2 mM DTT; 1% protease inhibitor cocktail; 0.2% NP-40) three times and washing buffer without detergent twice (100 mM Tris-HCL pH 8.0; 150 mM NaCl; 10% glycerol; 2 mM DTT; 1% protease inhibitor cocktail). Immunoprecipitated proteins were run in an acrylamide gel and stained with Coomassie blue, and bands covering each lane were excised with a scalpel and subjected to mass spectrometry analysis.

Mass spectrometry analysis was performed at the Proteomics facility of the Shanghai Center for Plant Stress Biology. Matching raw MS data to peptide sequences was performed using Mascot software with the annotated proteins from the *N. benthamiana* draft genome sequence v. 0.4.4 , which was obtained from the International Solanaceae Genomics Project (SOL) (https://solgenomics.net/). A decoy database was constructed with those protein sequences originated from randomized and reversed raw *N. benthamiana* protein sequences. The decoy hits were used to estimate the false-positive rate in the peptide-identification process. Because there was a large amount of potential protein-protein interactions (PPIs) identified from the AP-MS data, we used the MiST software (Mass spectrometry interaction STatistics) [3,23], removing the specificity criterion, to identify the high-confidence PPIs. High-quality MS data were filtered based on four criteria: (1) Protein threshold is larger than 99%; (2) minimum number of peptides is 1; (3) peptide threshold > 95%; (4) DECOY FDR is lower than 1%. The total spectrum count numbers of those identified proteins were obtained for further research. 

Before using the MiST score pipeline, the DECOY proteins and proteins detected with more than two peptides in the control sample (expressing free GFP) were removed. The retained bait-prey pairs were assigned scores with the MiST package, ranked based on the MiST scores (removing the specificity component), and the pairs with top 10% highest MiST scores were regarded as high-confidence PPIs. The TYLCV-*N. benthamiana* PPI network was visualized using Cytoscape [24]. Venn diagrams were drawn using InteractiVenn [25].

### 2.3. PPI Network Analysis

Because there is no experimental *N. benthamiana* interactome data, we inferred the connectivity of TYLCV-targeted *N. benthamiana* proteins and the properties of the TYLCV-*N. benthamiana* PPI network using those of the best homologs in *A. thaliana* in the existing *A. thaliana* interactome, which was constructed by combining PPIs from the TAIR10 database (http://www.arabidopsis.org/), the predicted interactome from Matthew Geisler [26], and the Plant Interactome Database (http://interactome.dfci.harvard.edu/A_thaliana/). This interactome dataset covers 9300 proteins and contains about 83,000 PPIs. 621 *A. thaliana* proteins were identified as the best homologs of the 728 *N. benthamiana* proteins included in the TYLCV-*N. benthamiana* interaction network. 436 out of these 621 target proteins were covered in the Arabidopsis interactome. The topology properties of the PPI network were calculated using an in-house R script. 77 target proteins had more than 50 connections in the network and were thus regarded as hub proteins. 

Based on the properties of their best homologs in Arabidopsis, seven *N. benthamiana* proteins that were targets of TYLCV had the potential to interact with other pathogen effectors [17]. The interaction/homolog network of TYLCV proteins, *N. benthamiana* proteins, Arabidopsis proteins, and other pathogens was constructed with Cytoscape [24].

### 2.4. Functional Analysis of TYLCV-Targeted Proteins

To understand the functional roles of the TYLCV-targeted proteins in *N. benthamiana*, gene ontology (GO) enrichment analysis was performed for the interactors of each TYLCV protein using topGO with the GO annotations included in the *N. benthamiana* draft genome v0.4.4 (https://solgenomics.net/). A GO term with a *p*-value < 0.05 was regarded as significantly enriched.

### 2.5. Evolutionary Analysis of TYLCV-Targeted Proteins

Orthologous relationships between *N. benthamiana* and potato (*Solanum tuberosum*) proteins were established by reciprocal BLASTP searches, and only reciprocal best hits were retained. The non-synonymous substitution/synonymous substitution rate (dN/dS) between a pair of orthologues was estimated using the maximum likelihood-based program PAML [27]. The substitution rates of all TYLCV-targeted proteins or TYLCV-targeted hubs were compared to those of 100 randomly selected *N. benthamiana* proteins. 

## 3. Results

### 3.1. Identification of Nicotiana benthamiana Proteins Associating with GFP-Tagged TYLCV Proteins

**Figure 1:**
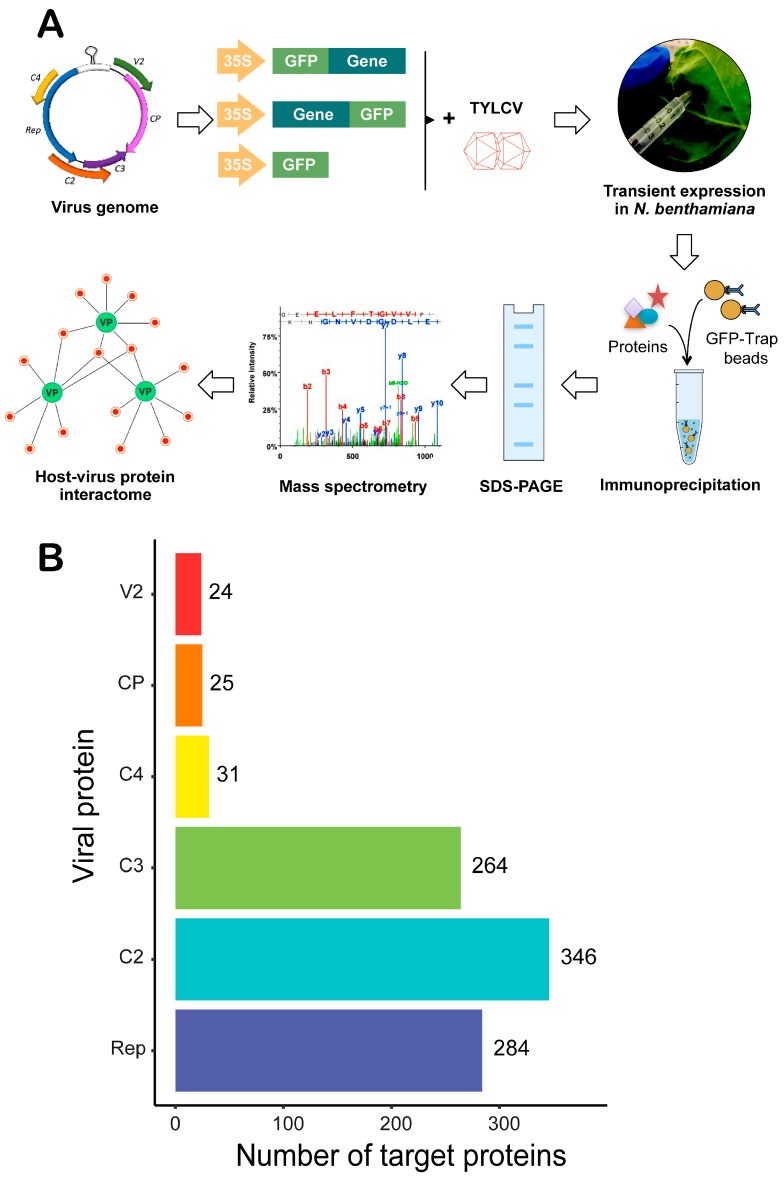
Schematic representation of the strategy followed to infer the TYLCV-*Nicotiana benthamiana* interaction network and number of *N. benthamiana* interactors identified. (**A**) Workflow of the strategy to identify interactors of TYLCV proteins in *N. benthamiana* by affinity purification-mass spectrometry analysis (AP-MS). TYLCV genes were cloned fused to GFP at their N- or C-terminus and transiently expressed in *N. benthamiana* leaves, together with a TYLCV infectious clone, by agroinfiltration. Two days later, total proteins were extracted and subjected to immunopurification using an anti-GFP resin. Immunopurified proteins were run in a sodium dodecyl sulfate-polyacrylamide gel electrophoresis (SDS-PAGE) gel, from which bands covering the entire lane were excised and sent for mass spectrometry analysis. Identified plant proteins co-purified with the viral GFP-fused proteins were used to infer the host-virus interactions network. In the example, viral proteins are depicted in green (VP), and interacting plant proteins are depicted in red. (**B**) Number of interacting plant proteins (referred to as target proteins) identified for each viral protein.

In order to define the TYLCV-*N. benthamiana* protein-protein interactome, we started by cloning all six genes from TYLCV (C1/*Rep*, *C2/TrAP*, *C3/REn*, *C4*, *V2*, and *CP*) fused to *GFP* at their N- or the C-terminus (Figure 1A). All viral genes were transiently expressed in *N. benthamiana* leaf patches through agroinfiltration in both N- and C-terminally tagged versions with the exception of *C4*, for which addition of an N-terminal tag disrupts one of its native localizations at the plasma membrane [28,29]. With the aim of mimicking the cellular environment during the viral infection, we co-infiltrated a TYLCV infective clone (GenBank identifier AJ489258; [30]), leading to efficient viral replication in the agroinfiltrated leaf, together with the binary vectors to express each GFP-tagged viral protein. Total proteins were extracted from the agroinfiltrated leaf patches two days after infiltration, and each viral protein was affinity purified using an anti-GFP resin. The resulting protein mixture was run in an acrylamide gel, and the presence of the immunopurified viral protein was checked by Coomassie staining (Supplementary Figure S1). Bands covering the entire lane were excised from the gel using a scalpel and subjected to mass spectrometry analysis to identify the proteins contained in the mixture (tagged viral protein plus associated host proteins) (Figure 1A).

The protein-protein interactions (PPIs) between the viral proteins and the co-immunoprecipiated host proteins were quantified using a modified version of a scoring system called MiST, previously applied to infer the HIV/human protein-protein interactome [3,23]. The original MiST score is a weighed sum of three parameters: protein abundance (measured by peak intensities from the mass spectrum), reproducibility (consistent presence in different replicates), and specificity (absence from the interactome of other viral proteins). For this work, considering that the whole viral proteome was present together with the tagged protein and that viral proteins might be part of common multi-protein complexes (as has been suggested, for example, for Rep and C3, which both participate in viral DNA replication), we eliminated the specificity component, giving rise to a scoring system composed of only two metrics, namely abundance and reproducibility. PPIs were then ranked according to their score, and the top 10% were retained as high-confidence interactors.

Following this approach, a total of 728 *N. benthamiana* proteins were identified with high confidence as part of the TYLCV-*N. benthamiana* interactome. More specifically, 65 proteins were identified as associated with GFP-Rep, 224 with Rep-GFP, and five were common to both; 296 proteins were identified as associated with GFP-C2, 129 with C2-GFP, and 79 were common to both; 52 proteins were identified as associated with GFP-C3, 217 with C3-GFP, and five were common to both; 31 proteins were identified as associated with C4-GFP; 21 proteins were identified as associated with GFP-V2, 12 with V2-GFP, and eight were common to both; eight proteins were identified as associated with GFP-CP, 21 with CP-GFP, and four were common to both. The total number of interacting host proteins identified as high-confidence interactors for each viral protein is represented in Figure 1B.

### 3.2. Inference of the TYLCV-N. benthamiana Protein-Protein Interaction Network and Its Properties

**Figure 2:**
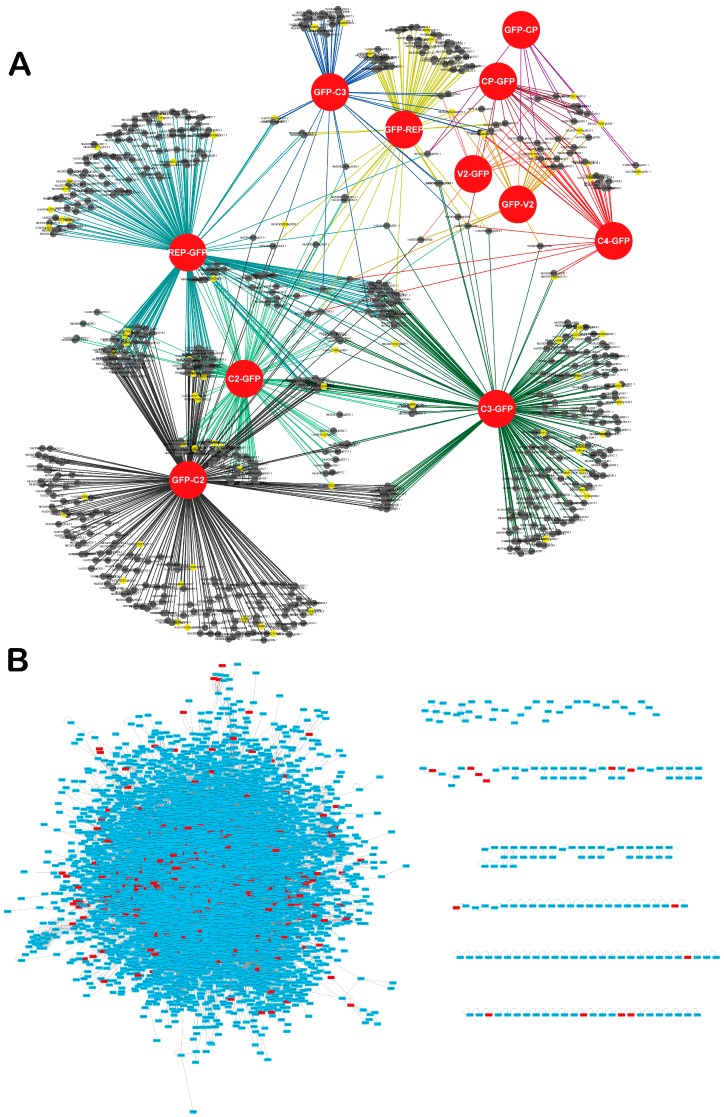
Network of TYLCV-*N. benthamiana* protein-protein interactions. (**A**) Network representation of TYLCV-*N. benthamiana* protein-protein interactions. Viral proteins (fused to GFP) are depicted as red circles. Best hits of Arabidopsis proteins identified as hubs in the Arabidopsis interactome are depicted as yellow nodes; non-hub proteins are depicted as black nodes. Lines (edges) indicate interactions between viral and host proteins. (**B**) Contextualization of the Arabidopsis best hits of the *N. benthamiana* interactors of TYLCV proteins in the Arabidopsis interactome. Best hits are depicted in red.

Once the subset of TYLCV-interacting proteins from *N. benthamiana* had been identified, we proceeded to (i) infer the PPI interaction network containing all viral proteins and all associated host proteins, representing direct or indirect interactions; and (ii) contextualize this network in the protein-protein interactome experimentally determined for *Arabidopsis thaliana* (hereafter referred to as Arabidopsis) [31]. The TYLCV-*N. benthamiana* PPI network inferred is shown in Figure 2A. Interestingly, a number of host proteins appear as associated with more than one viral protein (Figure 2A and Supplementary Figure S2), indicating that different viral proteins might be part of multi-protein complexes in the cell, and/or that these plant proteins are independently targeted by different viral proteins, which would suggest a high relevance of these targets in the interaction between plant and virus.

**Figure 3:**
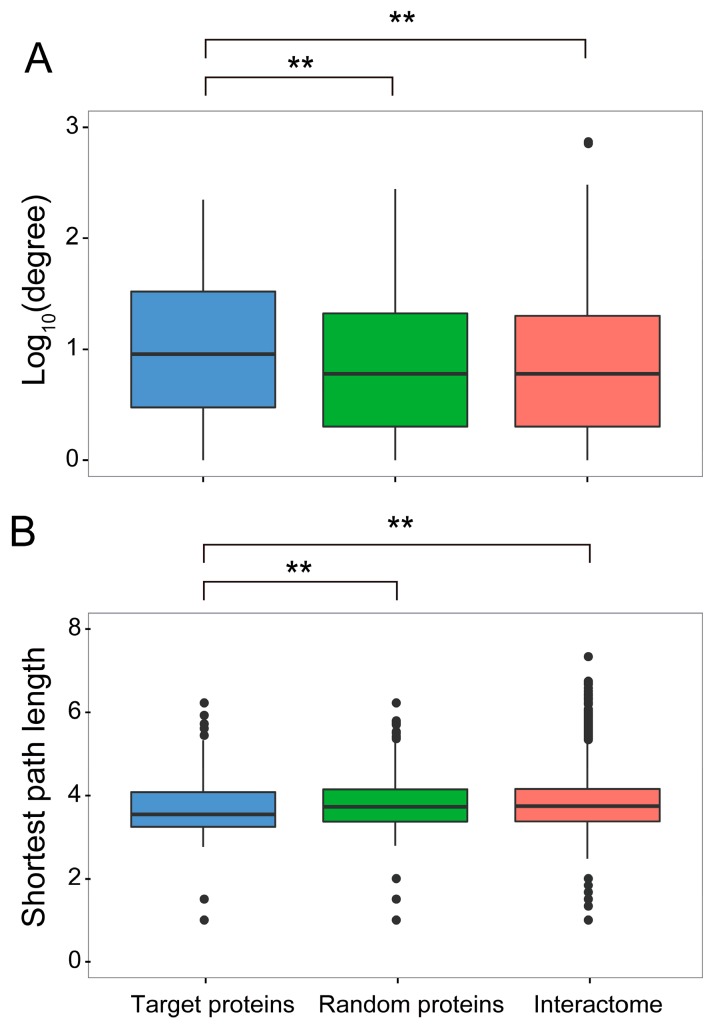
Global topological properties of the network. (**A**) Degree. (**B**) Shortest path length. These properties were calculated based on the 436 Arabidopsis best hits of the *N. benthamiana* interactors of the TYLCV proteins, termed target proteins, in the global Arabidopsis protein-protein interaction network. By comparison with control sets, both topology properties are significantly different between the Arabidopsis best hits and control sets (Mann-Whitney test; ** *p*-value < 0.01). A set of 200 randomly selected proteins, termed “random proteins” in the Arabidopsis interactome, as well as the whole interactome, were used as controls. The average degree exponent of target proteins was 0.964, while it was 0.838 in a random sample, and 0.837 in the whole network. The average shortest path of target proteins was 3.69, while it was 3.77 in a random sample and 3.80 in the whole network.

In order to contextualize the obtained PPI network in the global interactome of the host cell, we made use of the partial interactome available for Arabidopsis [31]. For this purpose, we first identified the best hits of the *N. benthamiana* interactors in Arabidopsis, therefore constructing an orthologous network; 621 proteins from Arabidopsis could be identified as best homologues of the 728 originally identified in *N. benthamiana*. Of these 621 proteins, 436 were contained in the Arabidopsis interactome (Figure 2B). Interestingly, we found that Arabidopsis best hits of TYLCV-interacting proteins from *N. benthamiana* were more connected in the Arabidopsis interactome than expected by chance (Figure 3A), indicating that they represented highly connected proteins or hubs. Indeed, 59 out of these 436 Arabidopsis proteins, corresponding to 77 homologous *N. benthamiana* proteins, had a degree >50.

Hub proteins are considered to play central roles in cell functions, and have been shown to be preferential pathogen targets in plants and animals [3,14,15,16,17]. Additionally, TYLCV-interacting proteins hold a position within the global interactome that allows them to connect with other cellular proteins through shorter paths than expected by chance (Figure 3B). These properties of the TYLCV/host PPI network indicate that local effects of the viral proteins on the cell proteome can have large-scale impacts in the cell rather quickly [32], resulting in major changes in cell functions upon a limited number of interactions.

### 3.3. TYLCV-Targeted Proteins Are Convergently Targeted by Other Pathogens

**Figure 4:**
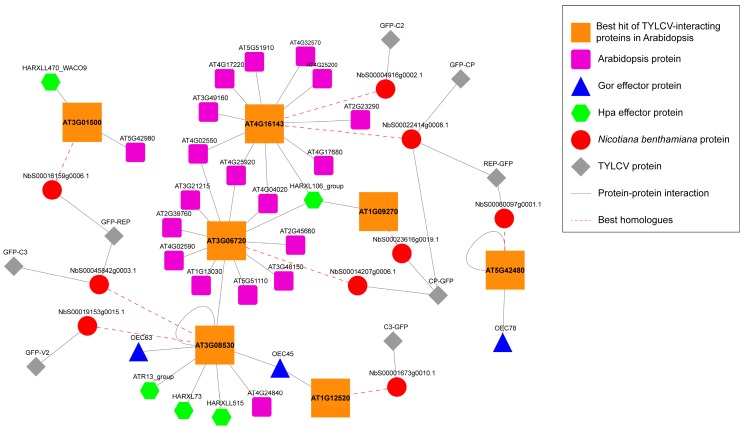
Network representation of the Arabidopsis best hits of the *N. benthamiana* interactors of TYLCV proteins convergently targeted by other pathogen effectors [15,17]. Gor: *Golovinomyces orontii*. Hpa: *Hyaloperonospora arabidopsidis*. *N. benthamiana* proteins interacting with viral proteins are depicted as red circles; TYLCV proteins are depicted as grey diamonds. Red dashed lines link *N. benthamiana* proteins and their best hit in Arabidopsis; Arabidopsis best hits of *N. benthamiana* proteins are depicted as big orange squares; other Arabidopsis proteins interacting with Arabidopsis best hits of *N. benthamiana* proteins are depicted as small purple squares. Effector proteins from *G. orontii* are depicted as blue triangles; effector proteins from *H. arabidopsidis* are depicted as green hexagons. Grey lines indicate protein-protein interactions.

Convergent targeting of Arabidopsis proteins has been shown for pathogens from three kingdoms of life (namely bacteria, fungi, and oomycetes) [15,17]. Interestingly, we found that the best hits of seven of these common targets in *N. benthamiana* also interact with TYLCV proteins (Figure 4). Four out of these seven proteins (those encoded by At1g0927, At1g12520, At3g06720, and At3g08530) showed a degree >50 and therefore could be considered hubs. The best hits of the proteins encoded by At3g06530 and At4g16143 were targeted by more than one viral protein, which may indicate convergent targeting among viral proteins or targeting by viral multi-protein complexes. A particularly interesting case was that of the product of At3g06530: this protein was independently targeted by three effectors from the oomycete *Hyaloperonospora arabidopsidis* (Hpa) and two effectors from the fungus *Golovimonyces orontii* (Gor), and two best homologues in *N. benthamiana* are targeted by three proteins from TYLCV, suggesting a crucial role in plant-pathogen interactions.

### 3.4. Functional Enrichment Analysis of the N. benthamiana Proteins Identified as Interactors of TYLCV Proteins

**Figure 5:**
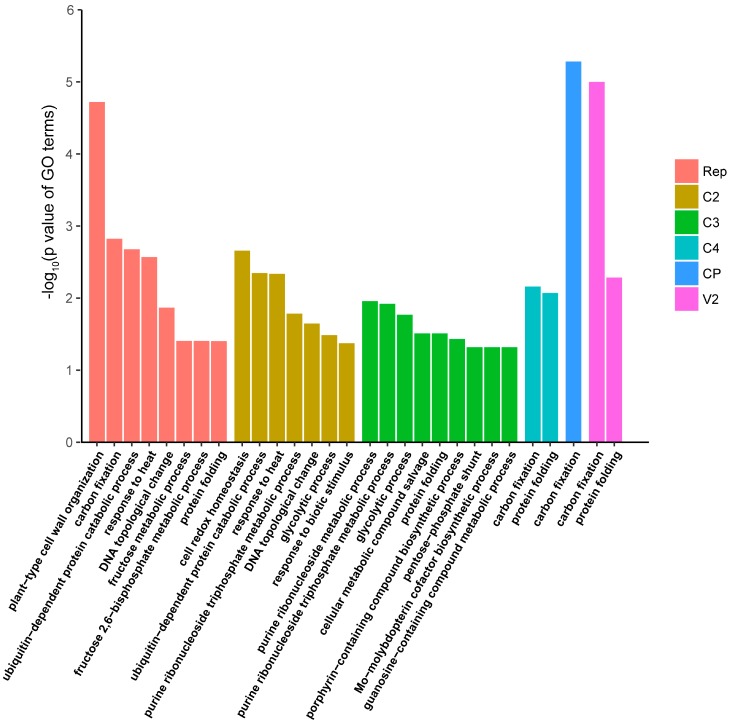
Functional enrichment analysis of *N. benthamiana* interactors of TYLCV proteins (Biological Process Ontology). A GO term with a *p*-value < 0.05 was regarded as significantly enriched. Over-represented GO terms in each subset of viral protein-interacting plant proteins are shown with different colours.

In order to gain insight into the cellular processes affected by each of the viral proteins, we performed functional enrichment analyses of their identified interactors in the host, with the aim of identifying GO terms (in the Biological Process, Molecular Function, or Cellular Component ontologies) over-represented in these subsets of proteins (Figure 5 and Supplementary Figure S3). All subsets of interactors showed over-represented GO terms in all three categories, with a *p*-value < 0.05. Interestingly, some of the enriched GO terms are in agreement with known functions of the corresponding viral proteins, and/or consistent with their localization. For example, the subset of Rep interactors shows an over-representation of proteins under the “DNA topological change” term; the subset of V2 interactors shows an over-representation of proteins under the “protein folding” term; the subset of C2 interactors shows an over-representation of proteins under the “ubiquitin-dependent protein catabolic process”. Given that Rep is involved in viral DNA replication [19], V2 localizes to the endoplasmic reticulum [33], and C2 has been shown to affect the regulation of ubiquitination-dependent protein degradation [34], interactors to which the aforementioned terms are assigned could contribute to the function of these proteins in the host cell.

### 3.5. TYLCV-Targeted Proteins Are Evolutionarily Constrained

**Figure 6:**
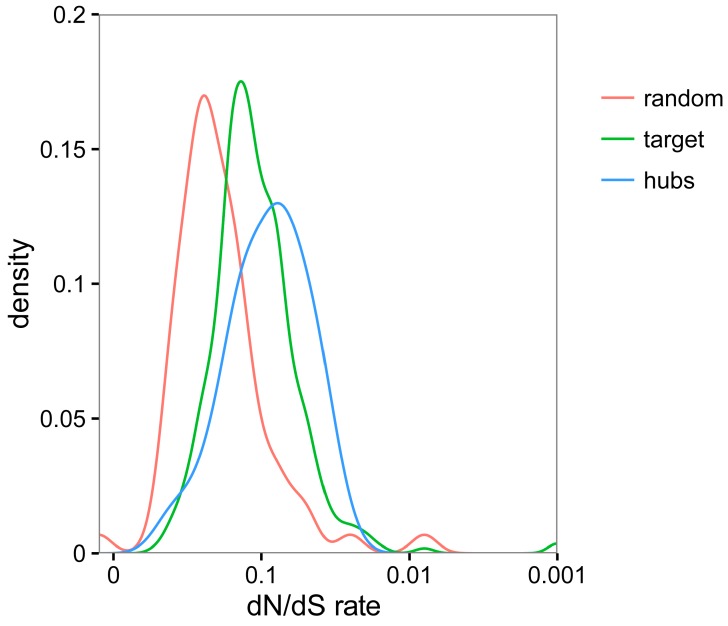
Comparative analysis of evolutionary rates (non-synonymous versus synonymous changes—dN/dS) between all TYLCV-interacting proteins (referred to as target; depicted in green), TYLCV-targeted hub proteins (depicted in blue), or randomly selected proteins from *N. benthamiana* (depicted in red) and the orthologous proteins in potato (*Solanum tuberosum*).

In order to investigate the evolutionary forces shaping the plant/virus interface, we decided to analyse the degree of conservation of the viral targets. We reasoned that, if TYLCV targets proteins with a central role in the cell physiology, the identified host interacting proteins might be highly conserved in plants; a high degree of conservation of viral targets has been described in the HIV/human interaction [3]. For this purpose, we compared the TYLCV-targeted proteins to a random subset of the *N. benthamiana* proteome in terms of their divergence pattern with respect to the orthologues from potato (*Solanum tuberosum*), another *Solanaceae* species that is not a host for TYLCV. Our results indicated that the TYLCV-targeted proteins identified in this work were more conserved than other proteins on average (Figure 6), indicating higher evolutionary constraints. As expected, the degree of conservation was even higher if only TYLCV-targeted hubs were considered (Figure 6).

## 4. Discussion

Given that viruses are intracellular parasites that rely on the host cell machinery for their functions, the inference of the host-virus protein-protein interaction network is a cornerstone to gain a comprehensive understanding of how the infection takes place or, in other words, what the molecular and physiological changes required for a successful viral invasion are. In this work, we used transient expression of tagged TYLCV proteins in TYLCV-infected leaf patches in the host *N. benthamiana*, followed by AP-MS, to identify plant proteins interacting with each of the viral proteins in infected cells. This approach yielded a set of high-confidence interactors, which we used to infer a network of virus/host protein-protein interactions. The validity of our approach was indicated by several findings. First, some of the interacting partners identified had been previously found, as exemplified by alpha-importins, known interactors of geminivirus CP and C2 [35,36]. Second, functional enrichment analysis yields categories that, at least in some cases, are in agreement with known functions and/or subcellular localization of the viral proteins. Third, the sets of interactors found for each protein are not fully overlapping, proving specificity and therefore discarding a prevalence of false positives due to the technical approach, which would be common to all. Additionally, analysis of the network revealed properties that were not expected by chance, such as higher connectivity for TYLCV-targeted proteins (discussed below). However, the limitations and shortcomings of the approach presented here need to be kept in mind. The fact that chloroplast proteins can be found in the lists of interacting host proteins for all viral proteins, although only geminiviral C4 has been described to localize in chloroplasts [28,29], suggests the presence of false positives in our network. Photosynthesis-related proteins are highly abundant in plants, and common contaminants in protein preparations; their identity as viral interactors has to be considered cautiously. On the other hand, not all viral interactors previously described are present in the interactome obtained in this work. This is most likely due to inherent limitations and biases of the method employed, since (i) the presence of the fused tag may interfere with some protein-protein interactions; (ii) not all host interacting proteins will be necessarily present in the tissue employed for viral protein expression in our experiments; and (iii) the probability of identifying a given interacting protein will depend on its intrinsic properties, such as amino acid sequence (which will determine the fragments produced after enzymatic digestion of the protein mixture, and their ability to be detected by mass spectrometry). Another aspect to bear in mind when interpreting the inferred network is that AP-MS results may represent both direct and indirect interactions, as opposed to, for example, yeast two-hybrid results, which represent direct interactions exclusively.

Interestingly, analysis of the network properties showed that the proteins targeted by the virus were highly-connected proteins, or hubs (Figure 3A). Preferential targeting of hub proteins has been previously shown for plant and animal pathogens [3,14,15,16,17], and suggests an evolutionary strategy that aims at maximizing the impact of protein-protein interactions, which are necessarily restricted by the limited number of viral proteins or other pathogen effectors. This idea is also supported by the finding that virus-targeted proteins are closely connected to other proteins (Figure 3B). Of note, some of these proteins were targeted by more than one viral protein (Figure 2A, Supplementary Figure S2), which may indicate multiple independent targeting or the formation of supra-molecular viral complexes. 

TYLCV-interacting host proteins can potentially play a role in the viral infection, and therefore may be good targets to explore in the quest for sources of resistance to the virus in crops. The fact that TYLCV targets hub and highly conserved proteins suggests that TYLCV is co-opting plant proteins with a central role in plant cell functions, most likely essential for survival of the host. In this scenario, knocking out the genes encoding TYLCV-targeted proteins may result in lethality, and analysis of their variability in different species or accessions may be a strategy with higher chances of success. Analysis of the conservation of these target proteins between TYLCV host and non-host plant species may point towards the most promising candidates, which would be worthy of investigation. 

Strikingly, the Arabidopsis best hits of some of the TYLCV-targeted proteins in *N. benthamiana* have been previously described as targets of effectors from pathogenic fungi and oomycetes (Figure 4). The interaction with proteins produced by TYLCV, an evolutionarily unrelated plant pathogen, further supports the proposed crucial role of these proteins in plant-pathogen interactions. As convergent targets of multiple independently evolved pathogens, these plant proteins could make enticing subjects for strategies to engineer broad-spectrum resistance in crops. Whether modification of these convergently targeted proteins may result in enhanced resistance to pathogens is an avenue worth exploring.

Our work represents a first step towards a global view of the virus/host protein-protein interaction interface. We selected a method to detect interactions in planta, and in the context of the infection, in an attempt to maximize the biological relevance of the identified targets. However, our approach provided only a snapshot of the interaction landscape during the infection; in order to expand our current view of the molecular events underlying viral invasion and generate a comprehensive model of the viral/host protein-protein interactions, additional parameters, such as tissue specificity and temporal dynamics, must be incorporated to future studies. Technical improvements allowing for isolation of native, untagged versions of the viral proteins from infected cells coupled to careful temporal dissection of the infection will pave the way for this much needed deepening of our understanding of the viral infection of the cell.

## Supplementary Materials

The following are available online at www.mdpi.com/1999-4915/9/10/275/s1, Figure S1: Example of Coomassie-stained gels of immunoprecipitated GFP-fused TYLCV proteins used for mass spectrometry analysis; Figure S2: Venn diagram of the identified plant interactors of each viral protein; Figure S3: Functional enrichment analysis of the *N. benthamiana* interactors of TYLCV Rep protein (GFP-Rep + Rep-GFP). Figure S4: Functional enrichment analysis of the *N. benthamiana* interactors of TYLCV C2 protein (GFP-C2 + C2-GFP). Figure S5: Functional enrichment analysis of the *N. benthamiana* interactors of TYLCV C3 protein (GFP-C3 + C3-GFP). Figure S6: Functional enrichment analysis of the *N. benthamiana* interactors of TYLCV C4 protein (C4-GFP). Figure S7: Functional enrichment analysis of the *N. benthamiana* interactors of TYLCV V2 protein (GFP-V2 + V2-GFP). Figure S8: Functional enrichment analysis of the *N. benthamiana* interactors of TYLCV CP protein (GFP-CP + CP-GFP); Table S1: List of *N. benthamiana* interactors of TYLCV proteins; Table S2: Arabidopsis best-hits of the *N. benthamiana* interactors of TYLCV proteins and their described interactors in the Arabidopsis proteome; Table S3: Functional enrichment analysis of the *N. benthamiana* interactors of TYLCV proteins (list of enriched GO terms and the corresponding genes—Biological Process, Molecular Function, and Cellular Component Ontologies); Table S4: Primers used in this work.
