# Supplementary material for: Inference of a Geminivirus−Host Protein−Protein Interaction Network through Affinity Purification and Mass Spectrometry Analysis
Source: Viruses. 2017 Sep 25;9(10):275. doi: 10.3390/v9100275 (PMC5691627; doi:10.3390/v9100275)
Supplement: Supplementary file 1 [file viruses-09-00275-s001.zip › Supplementary figures_edited.pptx]

## Slide 1
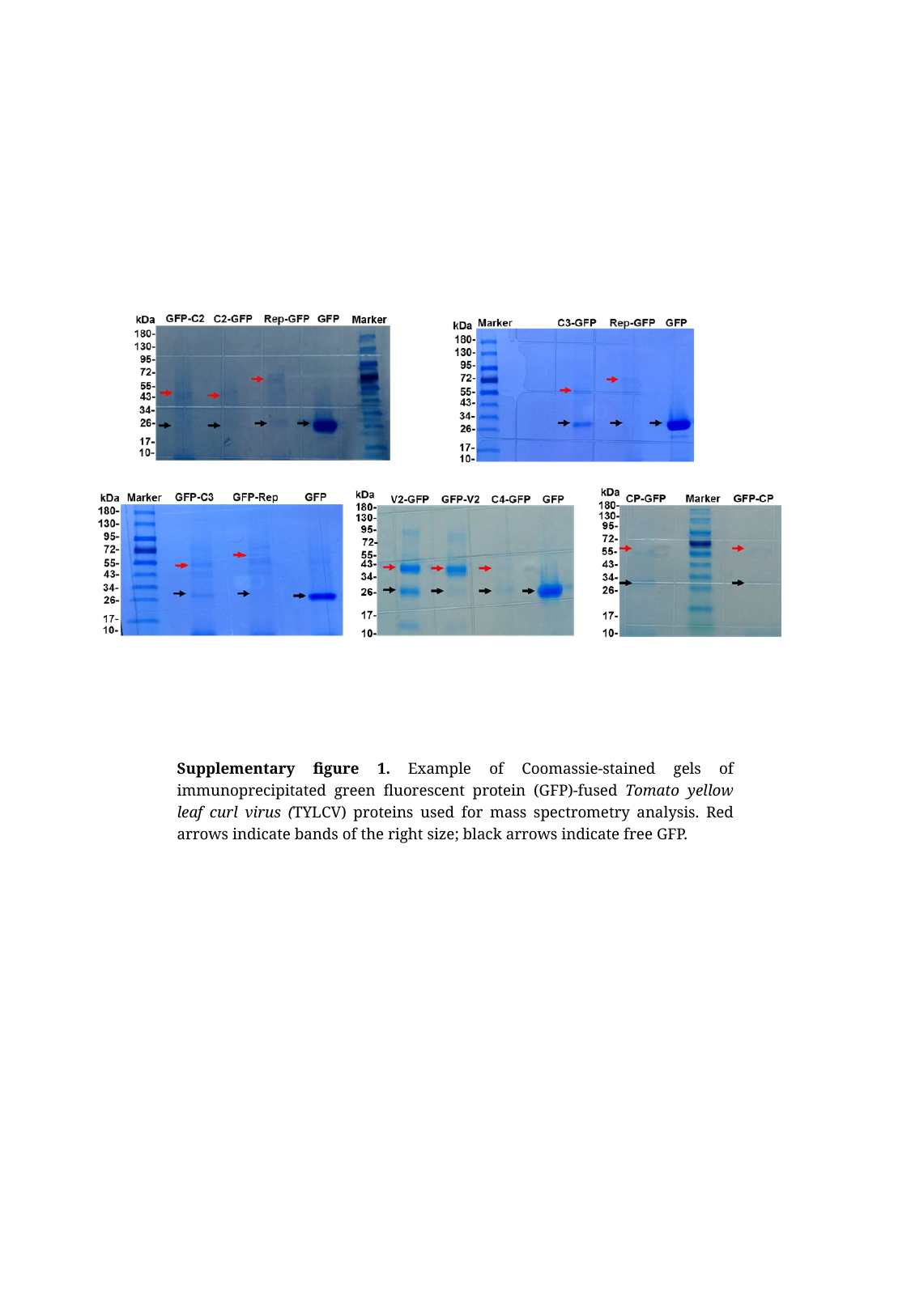

Supplementary figure 1. Example of Coomassie-stained gels of immunoprecipitated green fluorescent protein (GFP)-fused Tomato yellow leaf curl virus (TYLCV) proteins used for mass spectrometry analysis. Red arrows indicate bands of the right size; black arrows indicate free GFP.

## Slide 2
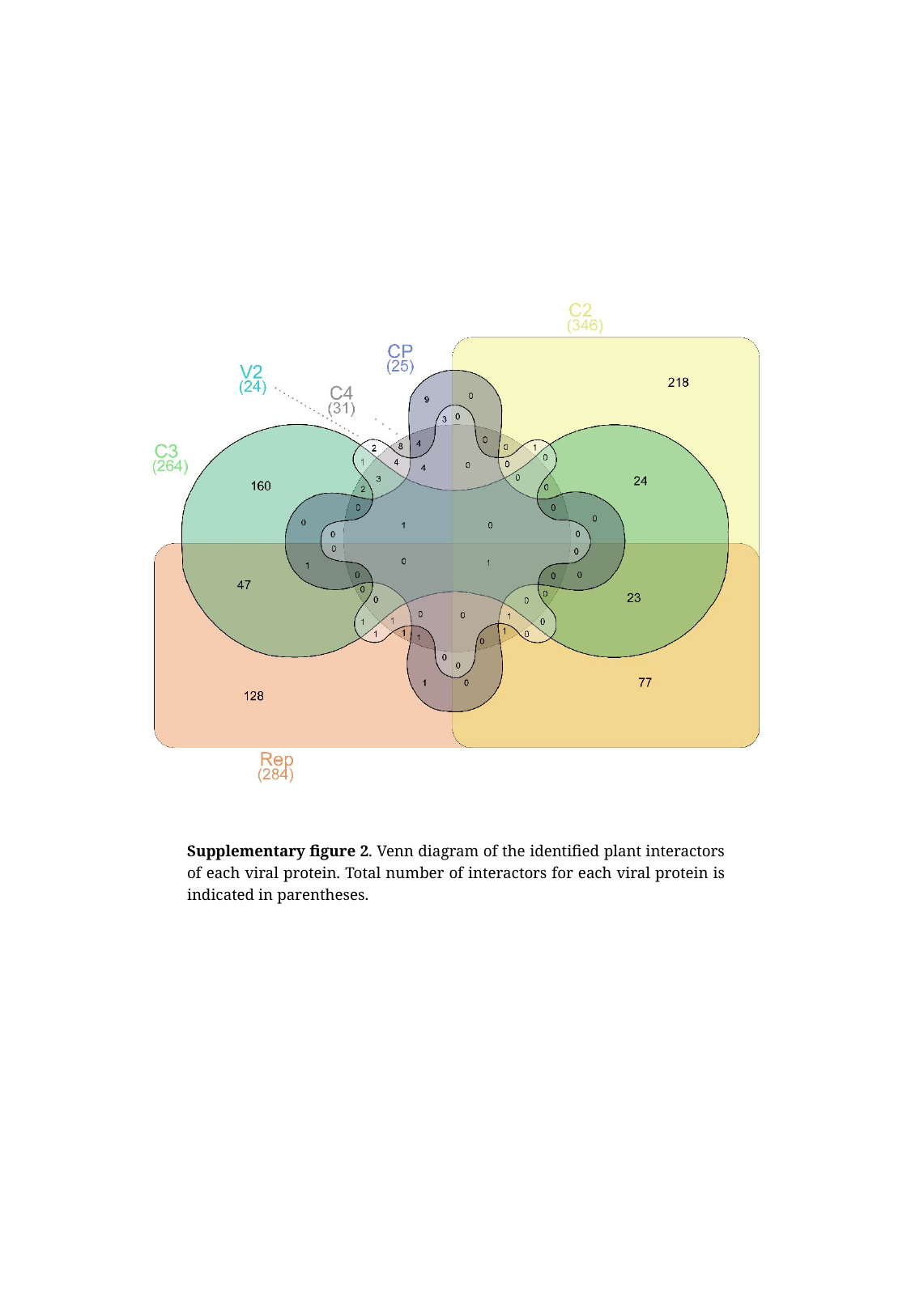

Supplementary figure 2. Venn diagram of the identified plant interactors of each viral protein. Total number of interactors for each viral protein is indicated in parentheses.

## Slide 3
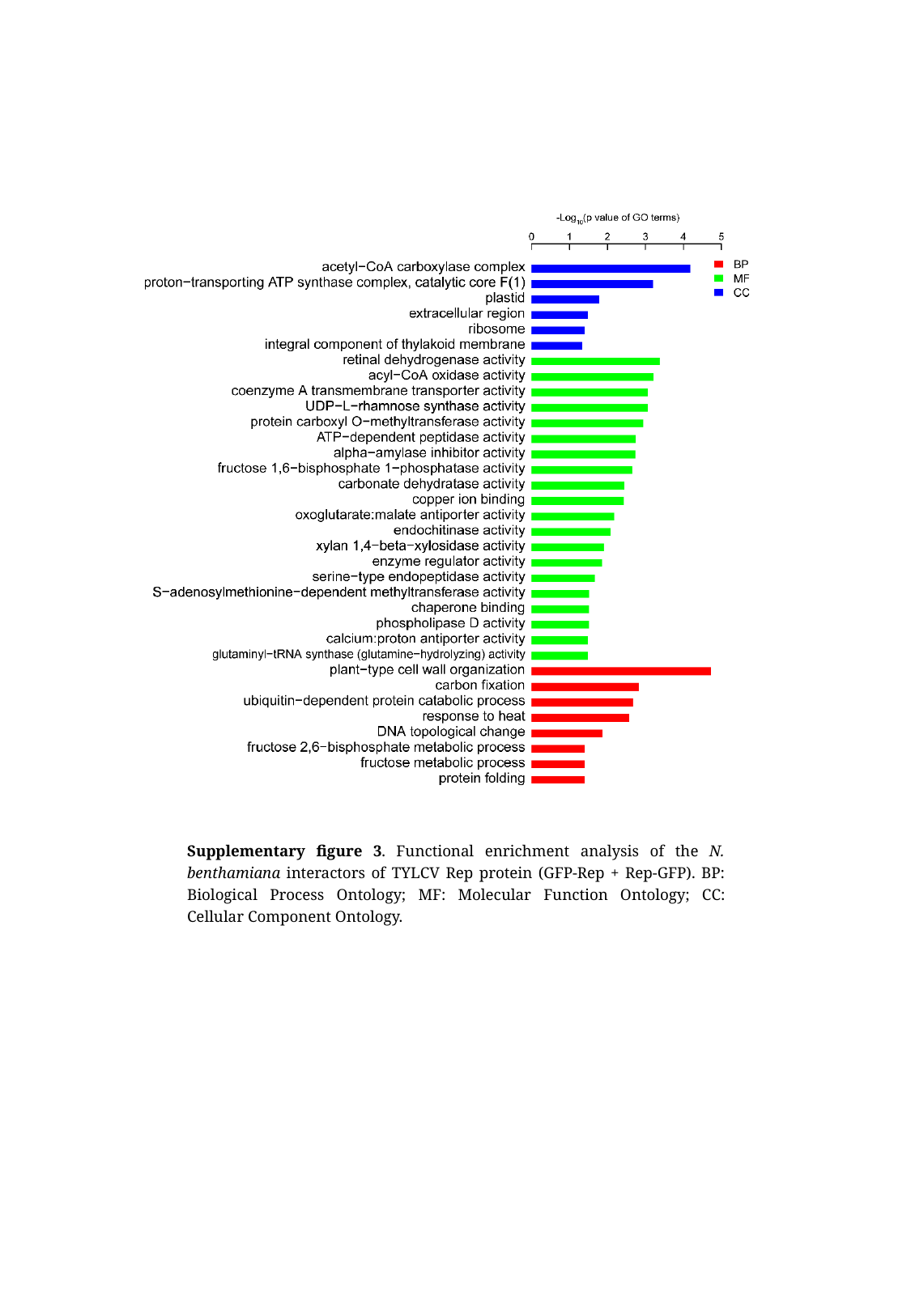

Supplementary figure 3. Functional enrichment analysis of the N. benthamiana interactors of TYLCV Rep protein (GFP-Rep + Rep-GFP). BP: Biological Process Ontology; MF: Molecular Function Ontology; CC: Cellular Component Ontology.

## Slide 4
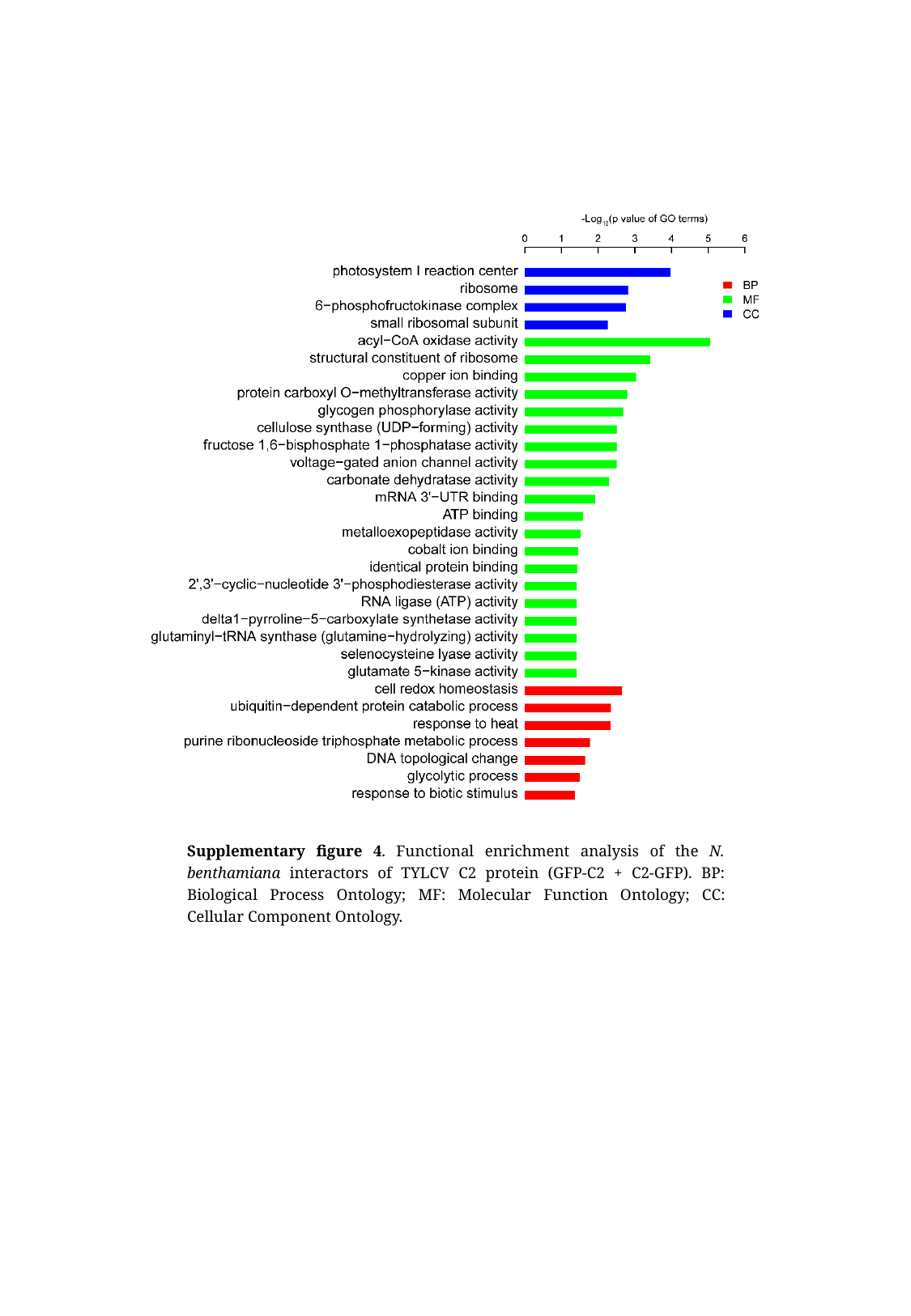

Supplementary figure 4. Functional enrichment analysis of the N. benthamiana interactors of TYLCV C2 protein (GFP-C2 + C2-GFP). BP: Biological Process Ontology; MF: Molecular Function Ontology; CC: Cellular Component Ontology.

## Slide 5
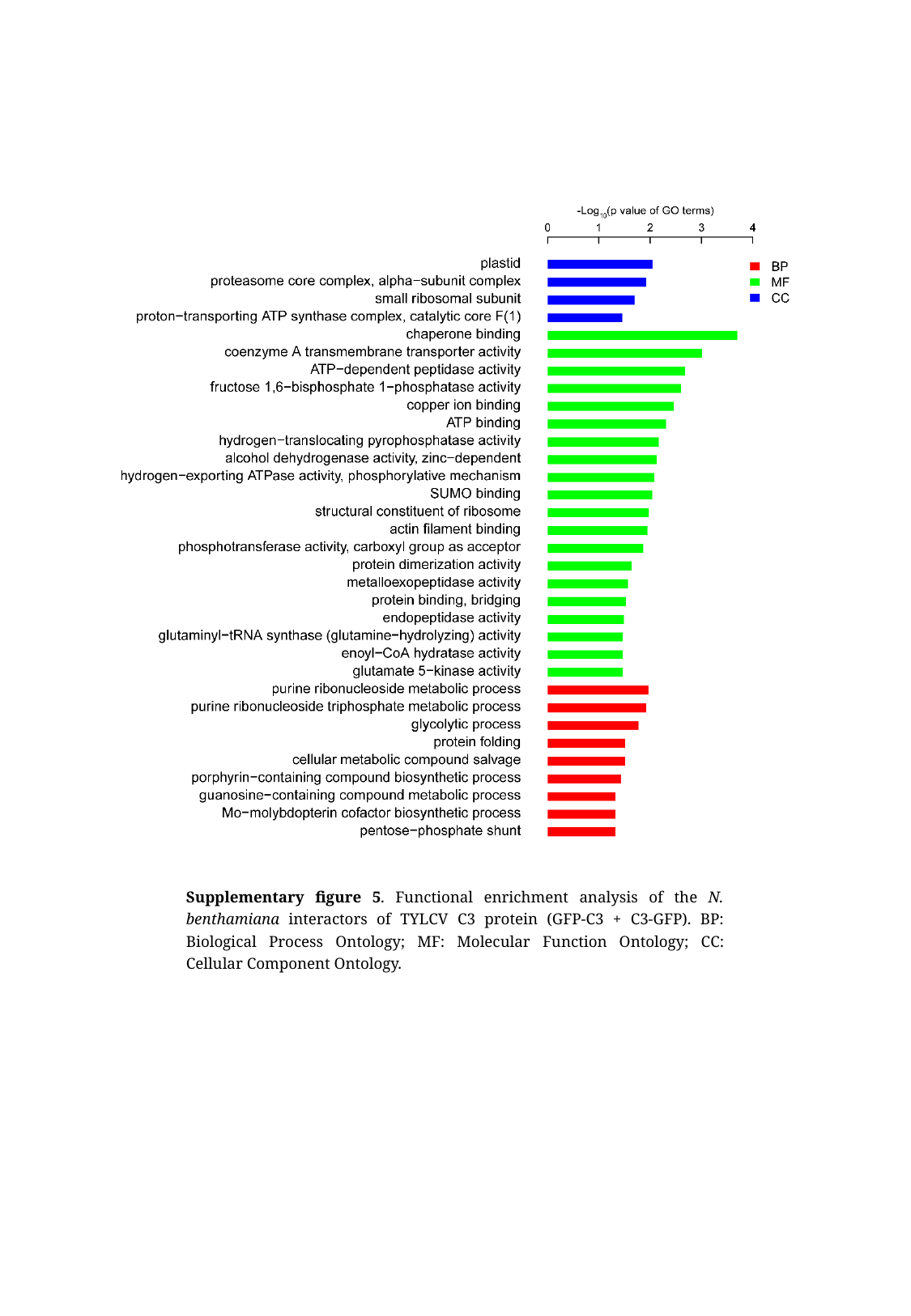

Supplementary figure 5. Functional enrichment analysis of the N. benthamiana interactors of TYLCV C3 protein (GFP-C3 + C3-GFP). BP: Biological Process Ontology; MF: Molecular Function Ontology; CC: Cellular Component Ontology.

## Slide 6
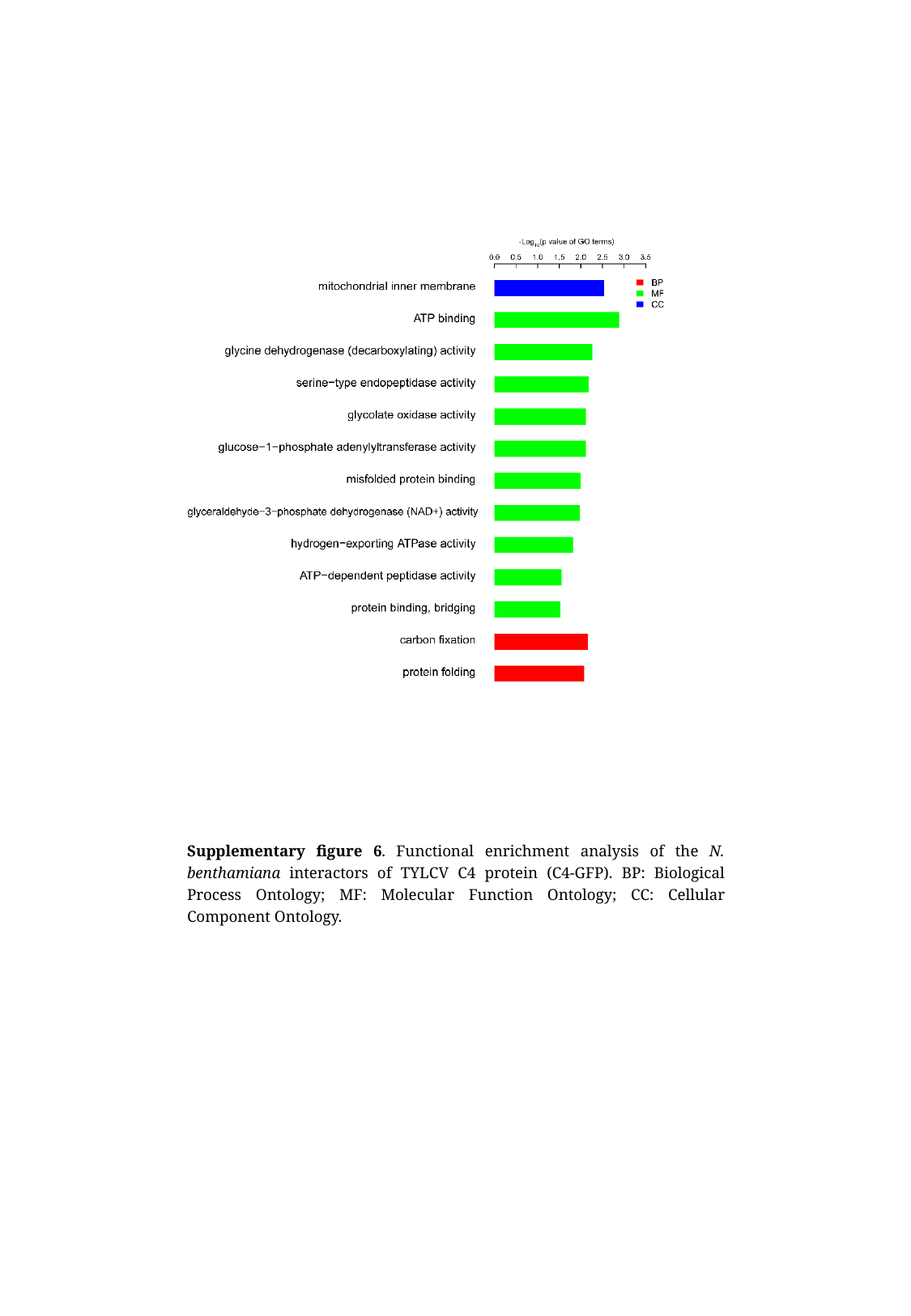

Supplementary figure 6. Functional enrichment analysis of the N. benthamiana interactors of TYLCV C4 protein (C4-GFP). BP: Biological Process Ontology; MF: Molecular Function Ontology; CC: Cellular Component Ontology.

## Slide 7
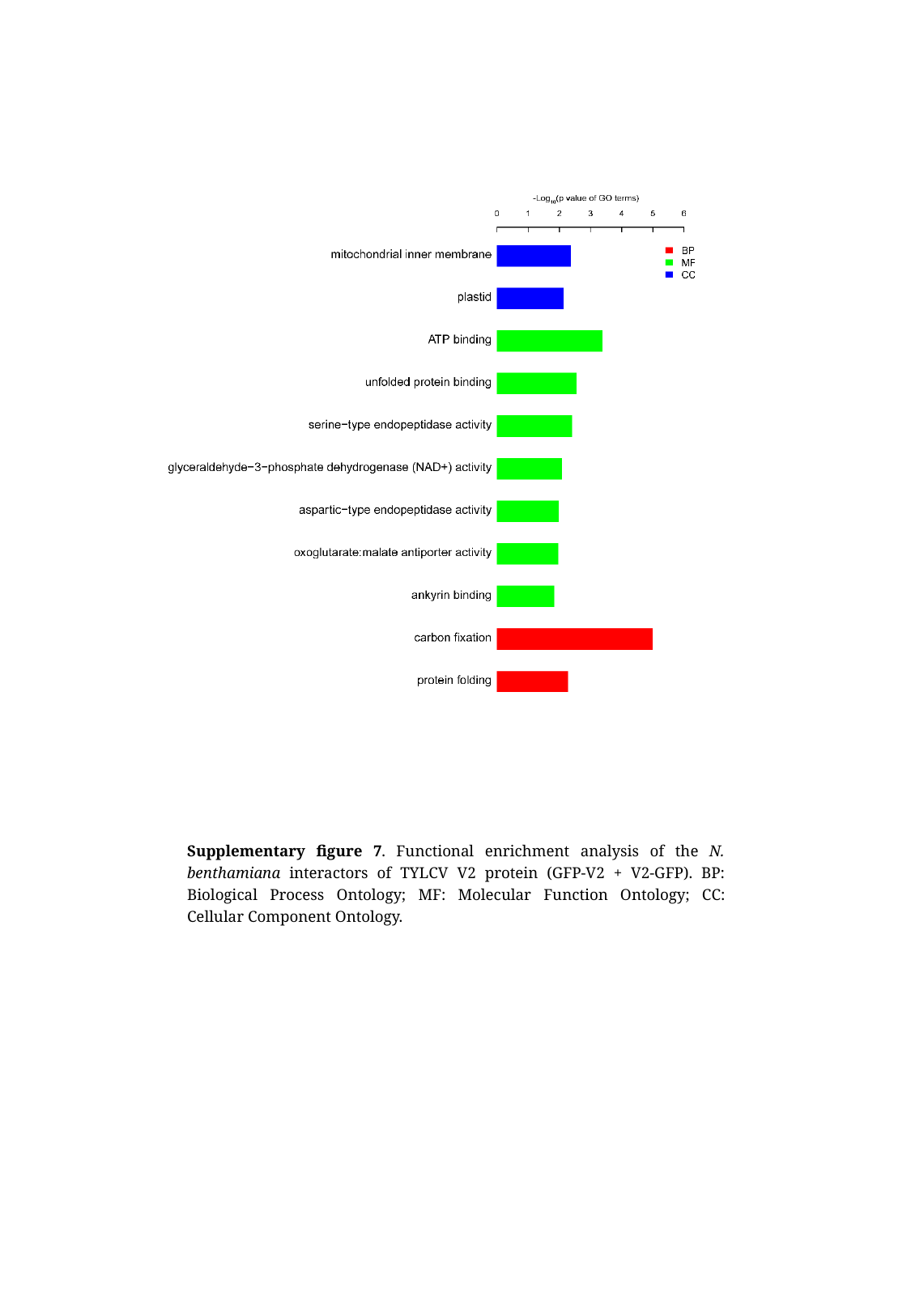

Supplementary figure 7. Functional enrichment analysis of the N. benthamiana interactors of TYLCV V2 protein (GFP-V2 + V2-GFP). BP: Biological Process Ontology; MF: Molecular Function Ontology; CC: Cellular Component Ontology.

## Slide 8
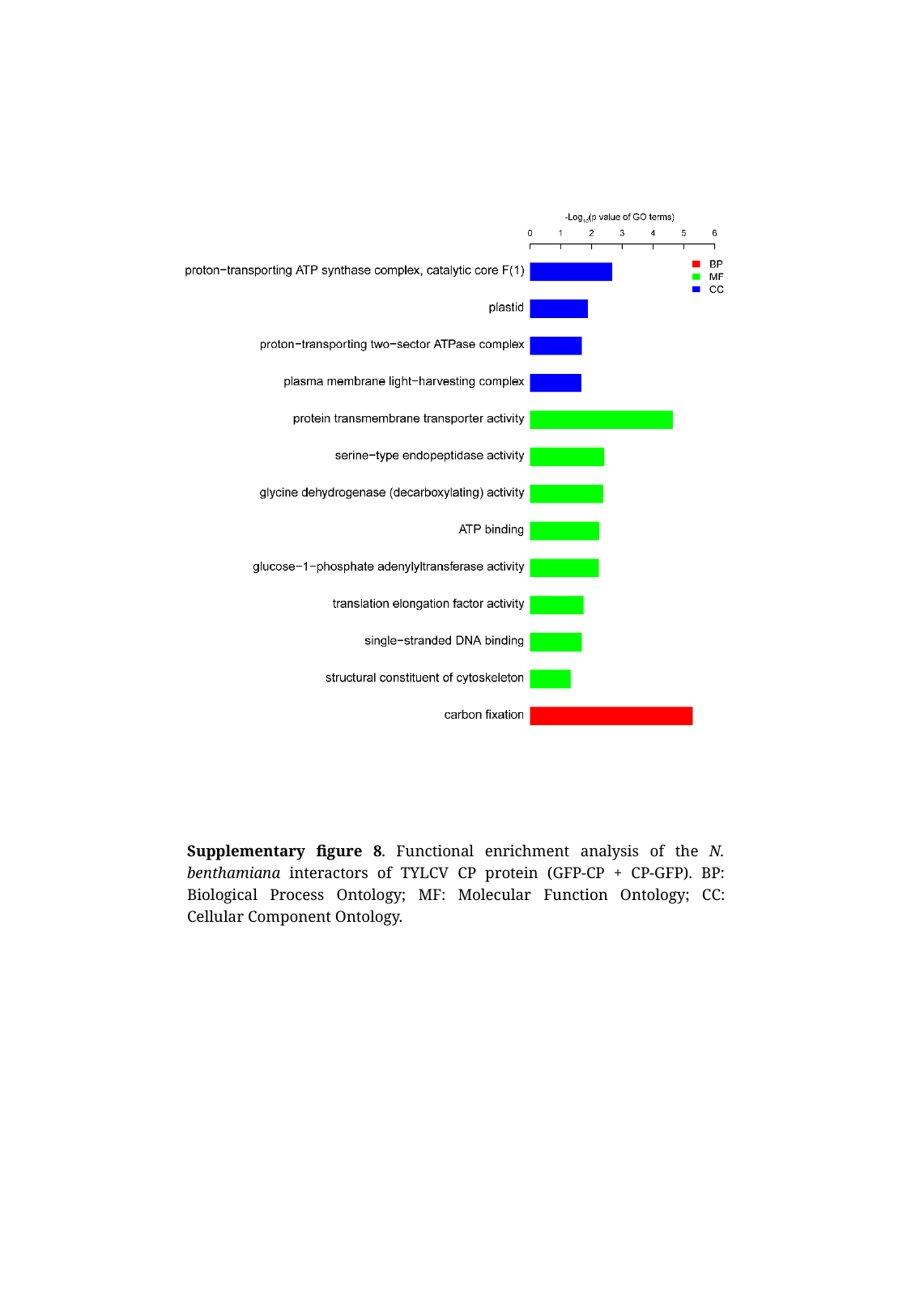

Supplementary figure 8. Functional enrichment analysis of the N. benthamiana interactors of TYLCV CP protein (GFP-CP + CP-GFP). BP: Biological Process Ontology; MF: Molecular Function Ontology; CC: Cellular Component Ontology.
