# Supplementary material for: Inference of a Geminivirus−Host Protein−Protein Interaction Network through Affinity Purification and Mass Spectrometry Analysis
Source: Viruses. 2017 Sep 25;9(10):275. doi: 10.3390/v9100275 (PMC5691627; doi:10.3390/v9100275)
Supplement: Supplementary file 1 [file viruses-09-00275-s001.zip › Supplementary table 4_Primers used in this work_edited.docx]

Supplementary table 4: Primers used in this work.

| **Primers used in this work** | |
| --- | --- |
| **Name** | **Sequence 5'-3'** |
| Rep-TOPO-F | CACCATGCCTCGTTTATTTAA |
| Rep-with stop codon-R | TTACGCCTTATTGGTTTC |
| C2-TOPO-F | CACCATGCAACCTTCGTC |
| C2- with stop codon-R | CTAAATACTCTTAAG |
| C3-TOPO-F | CACCATGGATTCACGCACAG |
| C3- with stop codon-R | TTAATAAAATTTATATT |
| C4-TOPO-F | CACCATGGGAACCACATC |
| C4- with stop codon-R | TTAATATATTGAGGG |
| CP-TOPO-F | CACCATGTCGAAGCGACCAG |
| CP- with stop codon-R | TTAATTTGATATTGAATC |
| V2-TOPO-F | CACCATGTGGGACCCACTTC |
| V2- with stop codon-R | TCAGGGCTTCGATAC |
| Rep-without stop codon-R | CGCCTTATTGGTTTC |
| C2-without stop codon-R | AATAGTGTTAAGAAATG |
| C3-without stop codon-R | ATAAAATTTATATTTTATATC |
| C4-without stop codon-R | ATATATTGAGGGCCTC |
| CP-without stop codon-R | ATTTGATATTGAATC |
| V2-without stop codon-R | GGGCTTCGATACATTC |
